# Radical cancer treatment is safe during COVID-19: the real-world experience of a large London-based Comprehensive Cancer Centre during the first wave

**DOI:** 10.1038/s41416-022-01909-0

**Published:** 2022-07-15

**Authors:** Beth Russell, Charlotte Moss, Maria Monroy-Iglesias, Graham Roberts, Harvey Dickinson, Kate Haire, Kathryn Innes, Bansi Mulji-Shah, Fiona Castell, Omar Al-Salihi, Mary Lei, Angela Francis, Bill Dann, Vikash Jogia, Hisham Hamid, Ben Challacombe, Ricard Simo, Stephanie Fraser, Charalampos Gousis, Elinor Sawyer, Eirini Tsotra, Jose Roca, Muhammad Khan, Debra Josephs, Deborah Enting, Mieke Van Hemelrijck, Victoria Harris, Saoirse Dolly

**Affiliations:** 1 Translational Oncology and Urology Research, School of Cancer and Pharmaceutical Sciences, King’s College London, London, UK; 2 South East London Cancer Alliance, London, UK; 3 Cancer Informatics and Data, Guy’s and St. Thomas NHS Foundation Trust, London, UK; 4 Guy’s Cancer, Guy’s and St. Thomas NHS Foundation Trust, London, UK

**Keywords:** Cancer, Cancer epidemiology

## Abstract

**Background:**

During the COVID pandemic, there was a paucity of data to support clinical decision-making for anticancer treatments. We evaluated the safety of radical treatments which were delivered whilst mitigating the risks of concurrent COVID-19 infection.

**Methods:**

Using descriptive statistics, we report on the characteristics and short-term clinical outcomes of patients undergoing radical cancer treatment during the first COVID-19 wave compared to a similar pre-pandemic period.

**Results:**

Compared to 2019, the number of patients undergoing radical treatment in 2020 reduced by: 28% for surgery; 18% for SACT; and 10% for RT. Within SACT, 36% received combination therapy, 35% systemic chemotherapy, 23% targeted treatments, 5% immunotherapy and 2% biological therapy. A similar proportion of RT was delivered in 2019 and 2020 (53% vs. 52%). Oncological outcomes were also similar to pre-COVID-19. The COVID-19 infection rates were low: 12 patients were positive pre surgery (1%), 7 post surgery (<1%), 17 SACT patients (2%) and 3 RT patients (<1%). No COVID-19-related deaths were reported.

**Conclusions:**

Whilst there were fewer patients receiving radical anticancer treatments, those who did receive treatment were treated in a safe environment. Overall, cancer patients should have the confidence to attend hospitals and be reassured of the safety measures implemented.

## Introduction

The provision of cancer services has been severely impacted by the outbreak of SARS-CoV-2 [1, 2]. Guy’s Cancer Centre in South East London treats ~8800 patients annually (including 4500 new diagnoses) [3] and is one of the largest Comprehensive Cancer Centres in the UK. The first coronavirus disease 2019 (COVID-19) positive cancer patient was reported at this site on February 29, 2020 [3]. In May 2020, we estimated the prevalence of COVID-19 in our cancer population to be 1.4% [3] highlighting the need for cancer patients to have the confidence to attend hospitals and be reassured that they will be treated in a COVID-19-managed environment.

However, the data from our Cancer Centre and its allied network of Hospitals covering South East London (population of 2.1 million people), supports previous reports that suspected cancer referrals declined sharply during COVID-19 wave 1 and wave 2, creating concern that cancer patients may eventually present with a later stage diagnosis (thereby missing an opportunity for curative treatment) [1, 4]. There was a 17.5% reduction in new cancer diagnoses for the time period March–September 2020 when compared with 2019— translating into an absolute estimate of 709 cases [5]. Furthermore, a comparison of April–September 2019 with the same period in 2020 revealed a decrease of 32% in cases identified via the 2-week-wait pathway (2096 vs 1423) and a 66% decline in screening-detected cases (198 vs 68), mainly due to the pausing of breast cancer screening services during wave 1. We noted a decrease of >20% for the following tumour types: urological (34%); breast (30%); sarcoma (29%); gynaecological (27%); skin (24%); lung (23%) as well as head and neck (20%). Consequently, we also observed a change in treatment modalities when comparing these two periods.

Having now undergone a second wave of COVID-19, it is important to review the outcomes and safety of these cancer treatments whilst balancing against the risks of COVID-19 infection and complications. Herein, we report on the patient and tumour characteristics as well as short-term clinical outcomes of those patients undergoing radical treatment (systemic anticancer treatment (SACT), surgery or radiotherapy (RT)) for their cancer during the first wave, as well as reporting the measures we undertook to mitigate risk, in order to help establish future clinical guidelines for the management of cancer patients in a SARS-CoV-2 pandemic.

## Methods

### Data sources

**Fig. 1:**
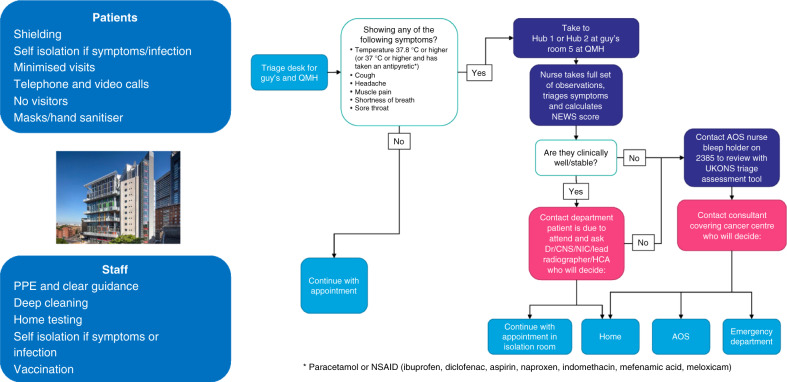
COVID-19 patient pathway at Guy’s Cancer Centre. The left panel details general provisions for staff and patients, and the right outlines the patient pathway.

Two data sources were utilised for this study. For the SACT and RT data, Guy’s Cancer Cohort, a research ethics committee-approved research database (Reference number: 18/NW/0297) of routinely collected clinical data of cancer patients at Guy’s and St Thomas’ NHS Foundation Trust (GSTT) [6]. Figure 1 outlines the COVID-19 patient pathway implemented at Guy’s Cancer Centre during the study period. The South East London Cancer Alliance (SELCA) obtained data for all patients that had radical cancer surgery through the pandemic via the SE London Cancer Surgery Hub. SELCA is 1 of 21 Cancer Alliances across England and works alongside and with its constituent Sustainability and Transformation Partnerships/Integrated Care systems/ICSs acting as the ‘cancer workstream’. There are three acute hospital trusts within the SELCA geography: GSTT; King’s College Hospital NHS Foundation Trust; and Lewisham and Greenwich NHS Trust.

### Study population

Data was extracted for all radical treatments (surgical, SACT and RT) for patients with solid malignancies; those undergoing palliative treatment and those with haematological cancers were excluded. Data on each treatment modality were extracted from two coinciding time periods: 1st March–8th September 2020 compared to the period in the same period in 2019. For SACT, 1st March–31st May 2019 was compared to 1st March–8th September 2020.

### Analyses

Information on the patients’ demographics and tumour characteristics were described and stratified by year of treatment (2019 vs. 2020). Short-term outcomes including length of stay and re-admissions were documented for surgical patients. For RT patients, information on dose and fractionation over the study period was noted. Further information extracted included the frequency of COVID-19 positive patients (defined by a positive PCR test) and mortality for all three treatment modalities. COVID-19 severity was defined according to the World Health Organisation criteria [7]. All data were compared using descriptive statistics.

## Results

During the period March–September 2020, 1552 cancer patients underwent radical surgery, 814 patients underwent radical RT and 1091 patients received a form of radical SACT (Table 1). In the same period in 2019, 2336 cancer patients underwent radical surgery, 1072 patients underwent radical RT and 819 patients received a form of radical SACT (March–May only). This equates to a reduction of 34% for surgery and 24% for radiotherapy compared to the same timeframe in 2019. There was a 18% reduction in radical SACT in 2020 (*n* = 668) compared to the same 3-month period in 2019 (*n* = 819). Using first-line cancer treatments as a proxy for newly diagnosed cancers, across all treatment types we estimate there to be a 23% decrease in the number of newly diagnosed patients undergoing treatment in 2020 compared to those diagnosed in 2019.

**Table 1:** Patient characteristics of those cancer patients receiving radical cancer treatment in 2019 and 2020.

	2020			2019		
	Surgery	SACT	RT	Surgery	SACT	RT
	(*n* = 1553)	(*n* = 1091)	(*n* = 814)	(*n* = 2336)	(*n* = 819)	(*n* = 1072)
Sex						
Male	894 (58)	265 (24)	343 (42)	1003 (43)	255 (31)	463 (43)
Female	659 (42)	826 (76)	471 (58)	1333 (57)	564 (69)	609 (57)
Age						
<50	312 (20)	250 (23)	129 (16)	441 (19)	190 (23)	130 (12)
50–59	373 (24)	324 (30)	206 (25)	264 (11)	211 (26)	199 (19)
60–69	402 (26)	260 (24)	239 (29)	504 (22)	205 (25)	305 (28)
70–79	349 (23)	227 (21)	188 (23)	558 (24)	183 (22)	310 (29)
≥80	117 (7.5)	30 (3)	52 (6)	569 (24)	30 (4)	128 (12)
Mean (SD)	61 (14)	58 (13)	62 (12)	63 (15)	59 (13)	65 (13)
Deprivation Index						
1	244 (16)	160 (15)	112 (14)	331 (14)	134 (16)	170 (16)
2	457 (29)	349 (32)	264 (32)	598 (26)	240 (29)	326 (30)
3	314 (20)	241 (22)	178 (22)	488 (21)	169 (21)	204 (19)
4	286 (18)	162 (15)	146 (18)	373 (16)	148 (18)	183 (17)
5	247 (16)	172 (16)	110 (14)	352 (15)	123 (15)	179 (17)
Missing	5 (<1)	7 (<1)	4 (<1)	194 (8)	5 (1)	10 (<1)
Ethnicity						
White British	413 (27)	339 (31)	283 (35)	689 (29)	353 (43)	520 (49)
White Other	135 (9)	91 (8)	48 (6)	182 (8)	57 (7)	72 (7)
Black Caribbean	17 (1)	44 (4)	21 (3)	54 (2)	32 (4)	30 (3)
Black African	26 (2)	41 (4)	26 (3)	45 (2)	29 (40)	50 (5)
Black Other	35 (2)	25 (2)	26 (3)	58 (2)	14 (2)	21 (2)
Asian	35 (2)	20 (2)	17 (2)	46 (2)	20 (2)	31 (3)
Mixed	44 (3)	11 (1)	5 (1)	23 (1)	12 (1)	9 (1)
Other	18 (1)	15 (1)	13 (2)	27 (1)	15 (2)	13 (1)
Unknown	830 (53)	505 (46)	375 (46)	1212 (52)	286 (35)	326 (30)
Comorbidities						
Hypertension	90 (8)	N/A	N/A	512 (22)	N/A	N/A
Diabetes mellitus	81 (5)	N/A	N/A	206 (9)	N/A	N/A
Lung conditions	88 (6)	N/A	N/A	59 (3)	N/A	N/A
Renal impairment	7 (<1)	N/A	N/A	142 (6)	N/A	N/A
Liver conditions	8 (1)	N/A	N/A	29 (1)	N/A	N/A
CVD	110 (7)	N/A	N/A	85 (4)	N/A	N/A
Unknown	853 (55)	N/A	N/A	512 (22)	N/A	N/A
Performance status						
0	582 (37)	244 (22)	482 (59)	279 (30)	248 (30)	591 (55)
1	391 (25)	173 (16)	286 (35)	452 (48)	307 (37)	376 (35)
2	103 (7)	17 (2)	43 (5)	178 (19)	42 (5)	93 (9)
3	8 (1)	1 (<1)	2 (<1)	16 (2)	2 (<1)	7 (<1)
4	6 (<1)	0 (0)	0 (0)	16 (2)	0 (0)	0 (0)
Unknown	463 (30)	656 (60)	1 (<1)	1395 (60)	221 (27)	5 (<1)

In 2020, there were more males than females in the surgical cohort (58% vs. 42%), whereas there were more females in both the SACT and RT cohorts (24% vs. 76% and 42% vs. 58%, respectively) (Table 1). In 2019, more females than males received all three treatment modalities. Whilst the age distribution was similar between treatment types, the mean age of the SACT patients was slightly younger compared to that of the surgical and RT patients in both years of study (2020: 58 vs. 61 and 62 years; 2019: 59 vs. 63 and 65 years respectively). The distribution of ethnicities was similar across the three treatment arms with the highest proportion of patients being White British.

Across all three treatment modalities, the most common tumour type was breast (2020: surgery 21%, SACT 50%, RT 40%; 2019: surgery 22%, SACT 39%, RT 39%) (Table 2). For both surgery and RT, urological malignancies were the second most common (2020: Surgery 20%, RT 24%; 2019: Surgery 17%, RT 23%). With regards to SACT, the second most common tumour type was GI in both years (2020: 26%, 2019 16%). In terms of the type of SACT, the majority of patients were on systemic chemotherapy in 2019 (59%) whereas in 2020, most patients were either on systemic chemotherapy (36%) or combination therapy (37%). The frequency of chemotherapy was reduced from 59% in 2019 (*P* < 0.0001), however, the frequency of combination therapy was increased from 7% in 2019 (*P* < 0.0001). The majority of patients were diagnosed less than a year prior to their treatment across all three treatment modalities.

**Table 2:** Cancer characteristics of those cancer patients receiving radical cancer treatment in 2019 and 2020.

	2020			2019		
	Surgery	SACT	RT	Surgery	SACT	RT
	(*n* = 1553)	(*n* = 1091)	(*n* = 814)	(*n* = 2336)	(*n* = 819)	(*n* = 1072)
Cancer type						
Breast	321 (21)	548 (50)	323 (40)	519 (22)	318 (39)	423 (39)
GI	201 (13)	175 (16)	77 (9)	371 (16)	210 (26)	96 (9)
Gynae	114 (7)	110 (10)	46 (6)	171 (7)	101 (12)	52 (5)
Head & neck and CNS	152 (10)	89 (8)	90 (11)	139 (6)	85 (10)	133 (12)
Liver	92 (6)	22 (2)	3 (<1)	116 (5)	0 (0)	1 (<1)
Plastics & skin	56 (4)	50 (5)	9 (1)	278 (12)	2 (<1)	9 (<1)
Thoracic	305 (20)	55 (5)	67 (8)	342 (15)	50 (6)	114 (11)
Urology	312 (20)	38 (3)	198 (24)	400 (17)	48 (6)	244 (23)
Other (e.g. sarcoma)	0 (0)	4 (<1)	1 (<1)	0 (0)	5 (1)	0 (0)
Cancer stage						
0/CIS	32 (2)	0 (0)	11 (1)	20 (1)	0 (0)	13 (1)
I	127 (8)	48 (4)	96 (12)	301 (13)	89 (11)	170 (16)
II	168 (11)	121 (11)	203 (25)	215 (9)	203 (25)	233 (22)
III	125 (8)	311 (29)	215 (26)	148 (6)	304 (37)	259 (24)
IV	74 (5)	130 (12)	63 (8)	92 (4)	118 (14)	99 (9)
Missing	1027 (66)	481 (44)	226 (28)	1560 (67)	105 (13)	298 (28)
Systemic treatment						
Systemic chemotherapy	N/A	388 (36)	116 (14)	N/A	485 (59)	98 (9)
Immunotherapy	N/A	54 (5)	0 (0)	N/A	12 (1)	0 (0)
Biological/targeted therapy	N/A	244 (22)	0 (0)	N/A	265 (32)	0 (0)
Combination therapy	N/A	405 (37)	0 (0)	N/A	57 (7)	0 (0)
Time since cancer diagnosis						
<3 months	681 (44)	221 (20)	390 (48)	1893 (81)	252 (31)	549 (51)
3–12 months	355 (23)	410 (38)	346 (43)	301 (13)	323 (39)	419 (39)
12–24 months	160 (10)	172 (16)	39 (5)	44 (2)	110 (13)	41 (4)
>24 months	87 (6)	288 (26)	38 (5)	93 (4)	134 (16)	63 (6)
Unknown	270 (17)	0 (0)	0 (0)	5 (<1)	0 (0)	0 (0)

The rate of surgery was similar across the specialities between the two time periods, except for a reduction in plastics 4% (compared to 12% in 2019; Table 2). The surgical parameters were comparable across the two study periods (Table 3). For example, the median surgical time was 120 min (IQR 73–183) in 2020 and 122 min (IQR 62–194) in 2019. The median theatre time was 195 min (IQR 138–263) in 2020 and 189 min (IQR 109–275) in 2019. There was a similar length of stay but a higher readmission rate in 2020 (11% vs 5% in 2019).

**Table 3:** Cancer treatment outcomes of those cancer patients receiving radical treatment in 2019 and 2020.

	First COVID-19 wave (2020)	Comparable period in 2019
SURGERY*		
ASA Grade III/IV/V	240 (22%)	N/A
Surgery time—min (median)	120 (IQR 73–183)	122 (IQR 62–194)
Theatre time—min (median)	195 (IQR 138–263)	189 (IQR 109–275)
ICU stay >24 h	155/1411 (11%)	133/1501 (9)
Pneumonia	55/946 (6%)	95/1501 (6%)
LOS—days	4	3
Re-admissions	36/335 (11%)	81/1501 (5%)
RADIOTHERAPY		
Dose reduction	52.0% of all courses of RT delivered were with radical intent	53% of all courses of RT delivered were with radical intent
Fractionation reduction	Supplementary Table 1	

^*^Denominator is varying as data is still being collected for some centres.

**Fig. 2:**
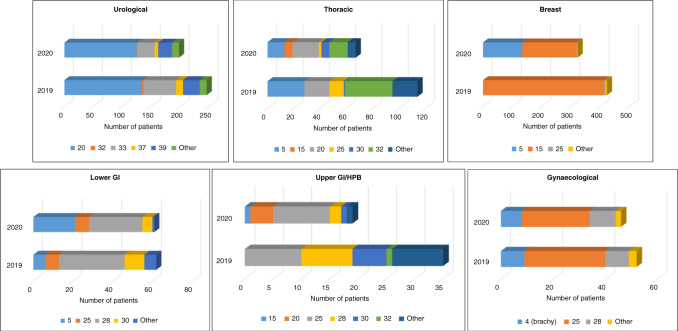
Radiotherapy fractionations for urological, thoracic, breast, lower gastrointestinal, upper gastrointestinal/hepatobiliary and gynaecological cancers in 2019 and 2020. Each colour block details the delivered fraction of radiotherapy in each tumour type.

In the RT cohort, there were 886 courses of radical RT delivered in 2020 out of a total of 1703, compared to 1141 courses (out of 2147) in 2019. Details of the doses are outlined in Supplementary Table 1 and displayed in Fig. 2. For breast and gastrointestinal cancers, there were more patients with hypofractionated RT schedules in 2020 compared to 2019. For example, 1/423 breast cancer patients received five RT fractions in 2019, compared to 134/323 patients in 2020. This is compared to 413 patients receiving 15 fractions in 2019, and 186 in 2020.

The proportion of patients who tested positive for COVID-19 was very low amongst all three treatment modalities (~1%) (Table 4). In the RT group, all COVID-19-positive patients had mild or moderate disease, whilst in the SACT group, 44% had severe disease. The proportion of patients who died in 2020 was also very low (varying from <1–2%) with no patients dying directly as a result of COVID-19 in the SACT or RT cohorts (information unavailable for surgical cohort). When comparing overall mortality from 2019, surgery had very similar mortality rates at both 30 and 90 days (0.5% and 1.8%, respectively) compared to 0.4% and 1.4%, respectively, in 2020 (Supplementary Tables 2 and 3).

**Table 4:** COVID characteristics and mortality status of those cancer patients receiving radical cancer treatment during the first wave of COVID-19 in 2020.

	Surgery	SACT	Radiotherapy
	(*n* = 1553)	(*n* = 1091)	(*n* = 814)
COVID status			
Negative	1534 (99%)	1075 (99%)	810 (99%)
Positive (30 days pre surgery)	12 (1%)	16 (1%)	4 (<1%)
Positive (30 days post surgery)	7 (<1%)	N/A	N/A
COVID severity			
Mild and moderate	N/A	9 (56%)	4 (100%)
Severe	N/A	7 (44%)	0 (0)
Death			
All-cause	19 (<1%)	10 (<1%)	14 (2%)
COVID-19	N/A	0 (0)	0 (0)

## Discussion

This study encompassed data from three treatment modalities collected during the first wave of the COVID-19 pandemic in the UK from a tertiary Comprehensive Cancer Centre. Results from this study suggest that our implementation of Covid-secure pathways and review of local treatment protocols meant that the treatment of cancer patients during this unprecedented time was safe with low levels of COVID-19 infection and low morbidity and mortality rates in line with that observed pre-pandemic.

Prior to the COVID-19 outbreak, there was a paucity of international guidelines addressing the management of cancer patients during the event of an infectious pandemic [8]. Early data from China suggested Covid-19 mortality in cancer patients was significantly higher than the general population [9]. As a result, our institutions working with regional and national counterparts had to rapidly adopt methods to prioritise those patients most in need of life-saving or prolonging interventions because capacity for delivering cancer treatments (particularly surgery) was reduced due to the escalating number of patients in acute hospital beds with COVID-19. Our centre, therefore, implemented COVID-19 minimal pathways whereby patients were advised to shield between hospital appointments and both self-isolate and provide a negative COVID-19 swab prior to admission for surgery [10]. Elective surgeries were stopped in the middle of March 2020 due to the risk of increased mortality for patients who contracted COVID in the peri-operative pathway. The South East London (SEL) Cancer Surgery Hub was established at the end of March 2020 to provide a COVID-minimal ‘green pathway’ for patients requiring time-critical elective cancer surgery. Patients were prioritised according to a SEL policy aligned to the national recommendations for the NHS on the prioritisation of surgical patients. SACT and RT were continued for most tumour types with radical treatment being prioritised and patients being evaluated on an individual basis to determine the risk vs. benefit of treatment. This prioritisation is reflected in our study whereby the readmission rates for 2020 surgical cases were higher than in 2019. This is indicative of the higher proportion of more complex surgeries taking place as a result of clinical prioritisation. For example, a greater proportion of the overall surgical caseload during 2020 were liver cases, compared to the corresponding period in 2019, which has high admission rates.

At GSTT, both SACT and RT are delivered in two stand-alone cancer centre buildings. Numerous strategies were employed to decrease footfall into these buildings to minimise risk to potentially immunocompromised patients, including personal protective equipment (PPE) made available to all staff with clear guidance on indications/ procedure; rapid adoption of telephone or video consultations; restriction of entry to patients only; temperature check and screening questions upon entry (patients and staff); masks and hand sanitiser applied upon entry to the building; asymptomatic Covid-19 swab testing of all patients (May–June 2020); isolation and testing protocol for suspected Covid-19 patients; and pathways to allow safe treatment of Covid-positive patients who were well enough to continue their treatment (typically patients in the midst of a course of RT).

Radical RT treatment typically involves patients attending for daily (Monday to Friday) treatment fractions for several consecutive weeks, with any interruptions or missed treatment fractions leading to a reduction in treatment efficacy. Therefore we rapidly reviewed treatment fractionation schedules to identify equivalent treatments which could be delivered in fewer visits without de-escalation of treatment intensity. Subsequent guidance was published online by the Royal College of Radiologists which was in keeping with our protocols. Figure 2 shows different RT fractionation schedules according to tumour type. Urological and gynaecological cancers show similar proportions of patients receiving different fractionations in 2019 vs. 2020. However, thoracic, lower gastrointestinal (GI) and upper GI/hepatopancreatobiliary (HPB) all show larger proportions of patients received longer treatment courses in 2019. Most dramatically, breast radiotherapy showed a large proportion delivered in 5 fractions in 2020 vs. 2019 following nationwide early adoption of this schedule following the presentation of results (but prior to publication) of the FASTforward trial of breast RT hypofractionation [11]. Another significant change to RT workload was the deferral of prostate cancer RT: all patients are commenced on neoadjuvant hormonal treatment for at least 3 months prior to prostate RT and this was continued whilst RT was deferred for patients to avoid the risk of hospital attendances. All deferred RT was delivered within the study period once COVID-19 rates had subsided.

Across the treatment modalities, the patient demographics were similar during the COVID-19 pandemic in terms of age, deprivation and ethnicity. Unlike previous reports suggesting that health inequality impacts access to COVID-19-related healthcare [12], we saw equal rates of radical treatment across the ethnic groups with Caucasian being the most commonly reported ethnicity. There was an increase in males proceeding to surgery in 2020 58% (vs. 43% in 2019, *P* < 0.0001), however, this may reflect an increase in urological surgery the majority being prostate surgery.

During the first wave of COVID-19 there was a reduction in radical treatments across the modalities., This was more pronounced for surgery (reduction of 34%) which in part is reflected by the reduction in surgical capacity during the first two months of the pandemic (April and May 2020). The SEL Cancer Surgery Hub was established at the start of wave one to prioritise patients according to national guidance and SEL decision framework to align with capacity available across the cancer network mainly in the independent sector. Patients identified as eligible for delayed surgery due to low risk of progression were mainly patients with breast and prostate cancer which are two of the largest volume cancer surgical modalities in SEL. Surgical activity in these specific tumour types was, however, increased during recovery from the first wave, from June 2020 onwards. The impact of COVID-19 on the delivery of SACT was less (18% reduction), although the frequency of chemotherapy was reduced from 2019 to 2020 (59–36%). Contrastingly, rates of combination treatment increased significantly to 37% in 2020 compared to 7% in 2019. This likely reflects the increased use or extension of neoadjuvant SACT/ RT treatment to bridge any delays to surgery and also the extended use of up-front hormone treatment in prostate cancer during the pandemic.

Results from this study show very low SARS-CoV2 infection and more importantly low mortality rates amongst our cancer patients who were undergoing radical treatment at the time of the UK outbreak. These low numbers are encouraging and suggest that the continuation of radical treatments across all three modalities was safe for our cancer patients. The relative cumulative drops in treatment from March to September are also considered low with treatment volumes increasing month on month (particularly in surgery) as the NHS adapted to treating patients via COVID-minimal pathways. Several studies have investigated outcomes of patients undergoing cancer treatment with concurrent COVID-19 disease, for example, a recent systematic review and meta-analysis concluded that active chemotherapy was associated with a higher risk of death compared to no active chemotherapy [13]. However, as so few patients were COVID-19 positive in our study it was not possible to quantify this risk. Notwithstanding, few studies have, like the current study, investigated the direct effects of the pandemic on the radical treatment of patients who do not have COVID-19.

Whilst several studies have looked at the clinical outcomes of patients undergoing surgery (particulary for head and neck cancer) [14, 15], there appear to be very few studies investigating patients undergoing RT and SACT during the COVID-19 pandemic. Therefore, the strengths of this study are both its novelty as well as the combined analysis of all three radical anticancer treatment modalities. Many publications around SACT are data from consortiums focusing on the COVID-positive population [16]. This study is unique in that it presents real-world data from one of the largest comprehensive Cancer Centres in the UK, and assesses the impact of COVID-19 in unselected patients receiving multimodality anticancer treatment.

A limitation to this study is the inclusion of just three months worth of data for SACT in 2019, although we are confident there were still sufficient numbers of patients within that timeframe to make reasonable comparisons. This limitation can be attributed to the lack of workforce available to collect and clean the data for this longer period. A further limitation is that we did not have the dosage information or data surrounding possible interruption of treatment for the SACT patients.

## Conclusion

Despite the immense challenges and uncertainties faced by clinicians, patients who underwent radical cancer treatments at our tertiary centre during the first wave of the UK COVID-19 outbreak did so in a safe COVID-19 managed environment. Comparison with pre-pandemic data showed a slight reduction in the overall numbers of patients being treated most likely due to: a sizable reduction in cancer referrals and diagnoses; prioritisations of patients most clinically in need of treatment; and the possible reluctance of some patients to come into hospital. Results from this study evidenced the need to maintain radical treatment for cancer patients during the second wave seen earlier this year (2021) as well as any future waves should they occur. Furthermore, our findings highlight that cancer patient should have the confidence to attend hospitals and be reassured of the safety measures implemented.

## Supplementary information

Supplementary Material
