# Supplementary material for: Radical cancer treatment is safe during COVID-19: the real-world experience of a large London-based Comprehensive Cancer Centre during the first wave
Source: Br J Cancer. 2022 Jul 15;127(7):1289–95. doi: 10.1038/s41416-022-01909-0 (PMC9284490; doi:10.1038/s41416-022-01909-0)
Supplement: Supplementary file 1 — Supplementary Material [file 41416_2022_1909_MOESM1_ESM.docx]

**Supplementary Material**

**Supplementary Table 1.** Radiotherapy fractionations employed by tumour site in 2019 vs. 2020 (1.3.19 -7.9.19 inclusive and 1.3.20-7.9.20 inclusive)

| **Cancer Type** | **No of RT fractions** | **Year** | |
| --- | --- | --- | --- |
|  |  | **2019** | **2020** |
| **Breast** | 5 | 1 | 134 |
|  | 15 | 413 | 186 |
|  | 25 | 4 | 1 |
|  | other | 5 | 2 |
| **GI (lower)** | 5 | 6 | 21 |
|  | 25 | 7 | 7 |
|  | 28 | 33 | 27 |
|  | 30 | 10 | 5 |
|  | other | 6 | 1 |
| **GI (upper/ HPB)** | 15 | 0 | 1 |
|  | 20 | 0 | 4 |
|  | 25 | 10 | 10 |
|  | 28 | 9 | 2 |
|  | 30 | 6 | 1 |
|  | 32 | 1 | 0 |
|  | other | 9 | 1 |
| **Gynae** | 4 (brachy) | 9 | 8 |
|  | 25 | 31 | 26 |
|  | 28 | 9 | 10 |
|  | other | 3 | 2 |
| **H&N** | 15 | 3 | 1 |
|  | 20 | 21 | 22 |
|  | 25 | 2 | 1 |
|  | 30 | 70 | 36 |
|  | 33 | 2 | 0 |
|  | 35 | 1 | 0 |
|  | other | 4 | 4 |
| **CNS** | 15 | 6 | 2 |
|  | 30 | 20 | 20 |
|  | 33 | 2 | 3 |
|  | other | 2 | 1 |
| **Skin** | 1 | 0 | 1 |
|  | 6 | 4 | 0 |
|  | 9 | 2 | 1 |
|  | 15 | 0 | 1 |
|  | 17 | 0 | 1 |
|  | 20 | 1 | 3 |
|  | 30 | 1 | 2 |
| **Thoracic** | 5 | 28 | 13 |
|  | 15 | 0 | 6 |
|  | 20 | 19 | 20 |
|  | 25 | 11 | 2 |
|  | 30 | 1 | 6 |
|  | 32 | 36 | 14 |
|  | other | 19 | 6 |
| **Urology** | 20 | 134 | 125 |
|  | 32 | 3 | 1 |
|  | 33 | 56 | 30 |
|  | 37 | 12 | 6 |
|  | 39 | 29 | 24 |
|  | other | 12 | 12 |
| **Other** | 25 | 0 | 1 |

**Supplementary Table 2.** COVID-19 infection and mortality data for surgery in 2020.

|  | **n** | **Total** | **Percentage of cases** |
| --- | --- | --- | --- |
| COVID + pre surgery (resulting in delayed surgery) | 12 | 1269 | 0.9% |
| COVID + post-surgery (Severity unknown - patient recorded) | 7 | 411 | 1.7% |
| Mortality 30 days (all cause) | 6 | 1549 | 0.4% |
| Mortality 90 days (all cause) | 19 | 1361 | 1.4% |
| Mortality rate (all cause) per 100,000 population 30 days | 387.3 |  |  |
| Mortality rate (all cause) per 100,000 population 90 days | 1396.0 |  |  |

**Supplementary Table 3.** COVID-19 infection and mortality data for surgery in 2019.

|  | **N** | **Total** | **Percentage of cases** |
| --- | --- | --- | --- |
| Mortality 30 days (all cause) | 12 | 2336 | 0.5% |
| Mortality 90 days (all cause) | 43 | 2336 | 1.8% |
| Mortality rate (all cause) per 100,000 population 30 days | 513.7 |  |  |
| Mortality rate (all cause) per 100,000 population 90 days | 1840.8 |  |  |
